# Role of AprA and pyocyanin from Pseudomonas aeruginosa on Staphylococcus aureus tolerance to silver

**DOI:** 10.1099/mic.0.001596

**Published:** 2025-09-03

**Authors:** Jakob Gorodetsky, Nadia Monych, Raymond J. Turner, Omid Haji-Ghassemi, Sean C. Booth

**Affiliations:** 1 Department of Biological Sciences, University of Calgary, Calgary, Alberta, Canada; 2 Department of Microbiology, University of Manitoba, Winnipeg, Manitoba, Canada

**Keywords:** AprA, metal-based antimicrobials, *Pseudomonas aeruginosa*, protease, pyocyanin, silver, *Staphylococcus aureus*

## Abstract

The opportunistic pathogens *Staphylococcus aureus* and *Pseudomonas aeruginosa* are often found together causing persistent infections where they exhibit complex interactions that affect their virulence and resistance to treatment. We sought to clarify how interactions between these organisms affect their resistance to the antimicrobial metal silver (AgNO_3_). As previous work showed that cell-free supernatant from *P. aeruginosa* enhances the resistance of *S. aureus,* we aimed to identify the exact factor(s) responsible for this increase. Using molecular weight cutoff filters and proteomics, we identified the protein AprA and pyocyanin as the responsible factors. Transposon-mediated disruption of *aprA* led to the production of supernatant which could not enhance the silver tolerance of *S. aureus*. These findings suggest that the protease AprA from *P. aeruginosa* plays an important role in increasing the tolerance of *S. aureus* to AgNO_3_ via in part by mediating the levels of pyocyanin which in turn reduces Ag^2+^ to detoxify it.

## Data Availability

All data generated or analysed during this study are included in this published article (and its supplementary information files).

## Importance

Alternative antimicrobials have seen a resurgence due to the rising levels of resistance to classical antibiotics by many pathogenic bacteria. Single-species cultures are highly susceptible to antimicrobial metals used in healthcare such as silver and copper, but microbes rarely exist in this state. *Pseudomonas aeruginosa* and *Staphylococcus aureus* are often associated with each other in chronic wound infections and people with cystic fibrosis, causing worse outcomes than either species alone. While they do compete with one another, these organisms also engage in complex and poorly understood biochemical interactions which influence each other’s growth under stressful conditions, such as exposure to metal antimicrobials. This warrants a need to understand and identify the molecules involved in mediating these interactions.

## Introduction

Multi-drug-resistant bacteria have seen a widespread increase in healthcare over the past 20 years [1,2]. As such, there has been a resurgence in the research of metals such as silver and copper as alternative antimicrobials [3,5]. Many infections involve more than one kind of bacteria, and these co-infections are more difficult to treat as interactions between co-infecting bacteria enhance resistance to antimicrobials [6,7]. Developing metal antimicrobials as clinical treatments thus requires investigating how susceptibility/resistance is influenced by interbacterial interactions.

Co-infection with multiple bacteria is common in people with cystic fibrosis, which affects 80,000 people per year worldwide [8]. *Pseudomonas aeruginosa*, one of the most prominent bacteria found infecting people with cystic fibrosis, is often found together with *Staphylococcus aureus*, and these two bacteria are also frequently found together in other kinds of infections including bronchitis and wounds [9,11]. Metal antimicrobials have shown promise for treating surface wounds as they can easily be impregnated into bandages [12].

We recently found that in simulated wound fluid (SWF), *P. aeruginosa* greatly enhances the resistance of *S. aureus* to both AgNO_3_ and CuSO_4_ (4× higher MICs) through secreted factors, which we identified by evaluating the effect of cell-free supernatant from spent culture media [13]. While several small-molecule metabolites (mostly amino acids (AA) and signalling molecules were identified which contributed to the enhanced resistance, these could not account for the entirety of the increase in resistance to silver. Here, we explored the high-molecular weight (MW) components of *P. aeruginosa* supernatant that increase the silver resistance of *S. aureus* and identify the secreted protease, AprA, as the major contributor. Additionally, we show that there is interplay between this protease and the phenazine pyocyanin that contributes to increased silver resistance in *S. aureus*.

## Methods

### Bacterial strains and culture maintenance

The strains utilized in this study were *P. aeruginosa* PAO1, and PCR-validated transposon mutants of candidate genes of protein targets, and *S. aureus* ATCC25923. All the strains used were obtained from −80 °C glycerol stocks from the Turner Laboratory. The strains were prepared by utilizing −80 °C glycerol stocks to streak lysogeny broth (LB) agar plates and grown at 37 °C. No strain had more than three passages from the originally obtained source. The use of *S. aureus* colonies for antimicrobial susceptibility analysis was conducted by suspending a single colony in liquid LB and incubating for 2 h at 37 °C, shaking at 150 r.p.m. After incubation, the inoculum was further diluted with LB to match a 0.5 McFarland standard. The inoculum was grown in SWF where 50% FBS and 50% peptone water (0.1 g l^−1^ peptone in 0.85% NaCl) were used.

### Metal preparation and storage

All metal salt stock solutions were prepared using distilled and deionized H_2_O (ddH_2_O) to give 50 mM AgNO_3_, and any further working stocks were prepared by diluting this primary stock with sterile ddH_2_O.

### Pyocyanin preparation and storage

Purified pyocyanin from *P. aeruginosa* P006 [≥98% (HPLC)] was purchased from Millipore-Sigma in its powder form. An aqueous stock of 10 mM pyocyanin was made by first diluting the pyocyanin in 10% ethanol and 90% ddH_2_O. This stock was further diluted into working stocks of 1 mM with ddH_2_O for experiments.

### Preparation of spent media

The spent media from *P. aeruginosa* strains were prepared by streaking single colonies from the LB plates and inoculating them into 25 ml of SWF or terrific broth media (where applicable). The two liquid inoculums were incubated at 37 °C and shaken at 150 r.p.m. overnight. The culture was collected into centrifuge bottles and centrifuged at 10,000 ***g*** for 30 min. The supernatants were filtered using 0.2 µm syringe filters to collect the spent media, which was further filtered using Amicon ultra-centrifugal MW filters of 3 kDa. These filters were centrifuged at 5,000 r.p.m. (~2,000 ***g***) in a swinging bucket rotor for 20 min. The resulting spent media concentrate that would be ‘above’ the MW cutoff was to be diluted in 20 mM Tris-Cl buffer solution (pH 8.0) to match the volume of the original spent media applied to the filter tube. The resulting spent media filtrate that would be ‘below’ the MW cutoff was not further manipulated.

### Analysis of protein composition of 50 kDa fraction

*P. aeruginosa* spent media were separated through systematic size fractionation using 3, 10, 50 and 100 kDa MW cutoff filters (Amicon, Merck Millipore Ltd). Each fraction was applied to the SWF media in the bioassay to identify which fraction gave rise to increased *S. aureus* metal resistance. The >50 kDa fraction was then further fractionated by FPLC either by size exclusion chromatography (SEC) on a superose-6 column (10 mM HEPES pH 7.5, 25 mM NaCl running buffer) or by ion exchange chromatography (IEC) on a MonoQ column (MOPS pH 8.0 with a linear gradient of NaCl, 0–1 M). In a second approach, spent media were first separated using a 60% ammonium sulphate precipitation, using the soluble fraction for subsequent SEC or IEC fractionation approaches. Fractions were dialysed or buffer exchanged using 30 kDa MW filters before being added to the SWF in the bioassay.

The active fraction was sent to the Southern Alberta Mass Spectrometry (SAMS) facility for LC-MS/MS analysis and protein identification. Eighteen proteins were found, but only those meeting 99% significance (Table S1, available in the online Supplementary Material) were followed up on. We focused on the higher abundance and top-ranked proteins and obtained transposon mutants of *P. aeruginosa* strain PAO1 from the verified library [14] for the genes of each of these top-listed proteins. Spent media from these mutants were used in the bioassay to identify which mutant gave rise to a loss of metal resistance phenotype.

### Pyocyanin extractions

Extractions were performed according to Essar *et al*. [15] with slight deviations. Three millilitres of culture supernatant were extracted using 3 ml of chloroform and subsequently extracted with 1 ml of 0.2 M HCl. The absorbance of this solution was measured at 520 nm.

### Silver susceptibility assay

A 0.5 McFarland standard inoculum was generated from an overnight culture of *S. aureus* in LB. This was further diluted 15-fold into a 96-well plate containing 2-fold serial dilutions of AgNO_3_ (193 to 0.754 µM) to obtain the lethal and sublethal challenge concentrations for the rapid bioassay for fractions.

A rapid bioassay was used throughout to assess fractions from spent media. A 96-well plate was prepared by adding SWF to the wells. The spent media, test fractions or compounds were then added to generate 16% test fraction and 84% SWF. To this, an appropriate amount of AgNO_3_ stock prepared in SWF was added to generate the challenge concentration. Inoculant was then added to give a final 30-fold dilution from an overnight culture of *S. aureus* culture diluted to 1.0 McFarland standard. The 96-well plate was then incubated at 35 °C for 10–24 h shaking at 120 r.p.m., and the OD of the plate was measured at the end time point at 600 nm using a PerkinElmer 2030 Victor X4 microplate reader. This provided an endpoint of the ability of *S. aureus* to grow under the stress of AgNO_3_ at a defined concentration.

The antimicrobial susceptibility assessment 96-well plates required corrections for initial turbidity before they were incubated due to the medium, inoculum and the higher metal concentrations at the start of the concentration series. Absorbances at 600 nm recorded at time 0 h were subtracted from the absorbances at time 24 h to standardize the growth measurement and determine the change in absorbance due to cell numbers.

### Statistical analysis

Statistics were performed using Prism version 10. Significant differences between different experimental groups for the antimicrobial susceptibility assays were determined using the Welch ANOVA test.

## Results

### Identification of protein component contributing to *S. aureus* metal tolerance

**Fig. 1.**
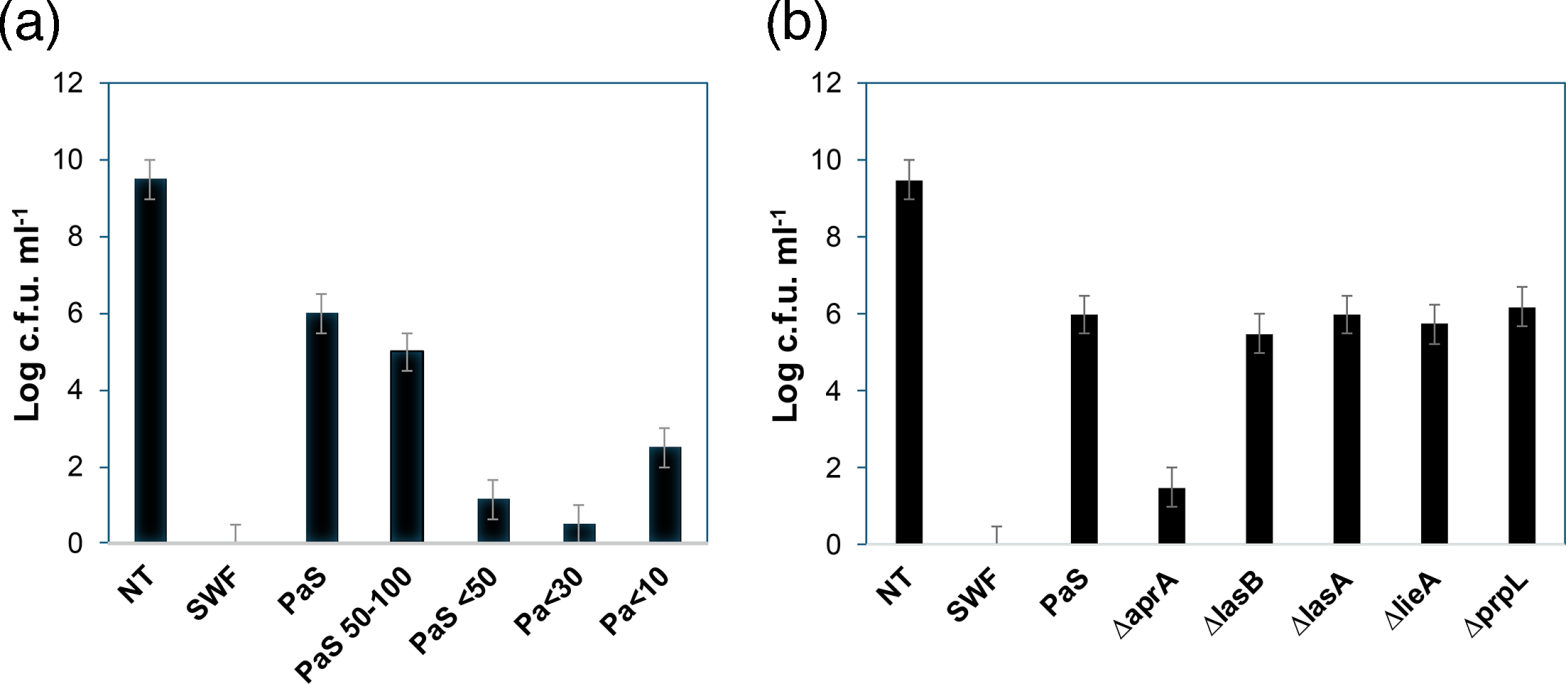
*S. aureus* growth after exposure to 250 µM AgNO3 with and without the addition of spent media fractions from *P. aeruginosa*. The log c.f.u. ml^−1^ of *S. aureus* was determined after exposure to 250 µM AgNO3 for 8 h at 37 °C with 150 r.p.m. shaking. NT represents the culture not treated with either silver or SWF. SWF NC represents growth in SWF without silver challenge. SWF represents no spent media added. PaS represents the secreted compounds from the planktonic growth of *P. aeruginosa* without any fractionation. (a) Effect of fractionation of spent media. PaS fractions include 50–100 kDa MW fraction, less than 50 kDa, less than 30 kDa and less than 10 kDa, respectively. (b) Using spent media (16% of culture media) from *P. aeruginos*a transposon mutants of selected genes. Values and error bars represent the average and sd of three biological replicates with two technical replicates each.

Our previous study [13] focused on molecules in the <3 kDa range that enhanced *S. aureus* metal tolerance. However, we also observed higher MW fractions of the *P. aeruginosa* spent media contributing to the phenotype. Using MW cutoff filters of 10, 30, 50 and 100 kDa, we found that the 50–100 kDa fraction increased the silver tolerance of *S. aureus* when added to the SWF media (Fig. 1). This figure also shows some silver tolerance that comes from the <10 kDa fraction molecules identified in the initial study [13]. We heat treated these fractions at 95 °C for 30 min and found that the activity was lost. Since the effector in this fraction could be inactivated by heat, it was expected to be proteinaceous, so it was further fractionated by chromatography.

The highest activity fraction from SEC was then evaluated by proteomic LC-MS/MS, identifying GroEL, LasB, AprA, PrpL, LasA and LieA as being present in this fraction (Table S1). From these, GroEL is a 60 kDa chaperone that often shows up in proteomics under stress-induced conditions and thus was not considered to be the effector. The others are all secreted proteases. As the AA serine and threonine were found to increase the metal tolerance of *S. aureus* [13], it seemed conceivable that secreted proteases could be cleaving proteins or polypeptides within the SWF, increasing AA levels in the media of the closed incubation system and contributing to metal tolerance. To determine which of these proteases was contributing to the increased metal tolerance, we screened single transposon mutants of the genes for each of these proteins. Only the spent media from the *∆aprA* strain did not increase the tolerance of *S. aureus* to silver (Fig. 1b). Thus, the role of AprA (enzyme name: serralysin), an alkaline protease which binds Zn^2+^ and Ca^2+^ [16], in silver resistance of *S. aureus* was thus subjected to further enquiry.

**Fig. 2.**
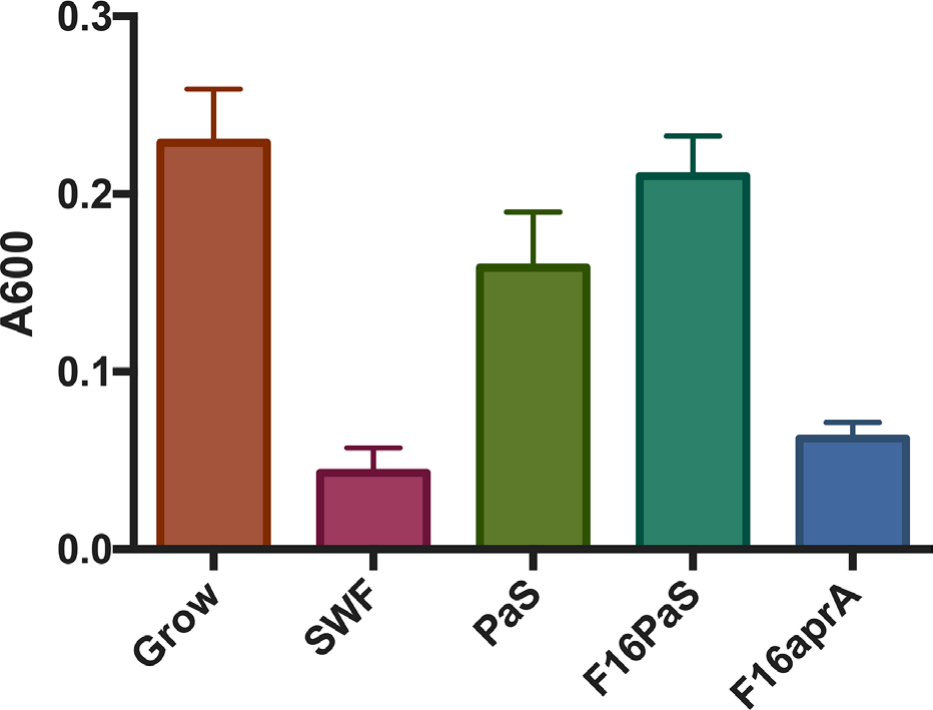
Protein complementation validating the silver tolerance effector. Fraction 16 from IEC demonstrates that only WT source (F16PaS) compared with ∆*aprA* (F16aprA) provides silver resistance. Growth evaluated by absorbance at 600 nm. Cultures grown for 8 h in the presence of 60 µM AgNO_3_. Average of three biological replicates with sd.

The role of AprA in silver resistance was further confirmed using partially purified proteins from the supernatant of the WT and compared with the *∆aprA* mutant (Fig. 2). We were able to partially purify AprA by ammonium sulphate precipitation (Figs S1–S3) followed by MonoQ IE, leading to an AprA-enriched fraction. Fraction 16 from the MonoQ column was unique to WT PAO1, and this peak is missing in the chromatogram from *∆aprA* strain of *P. aeruginosa* (Fig. S4). At this point, the AprA was estimated to be ~85% pure from SDS-PAGE analysis (Fig. S5). The addition of fraction 16 from *P. aeruginosa* spent media (F16PaS) to fresh SWF provided silver tolerance, whereas fraction 16 from the *∆aprA* strain of *P. aeruginosa (F16aprA*) did not provide the metal tolerance of *S. aureus* (Fig. 2), supporting that it was the protein AprA that increases the silver tolerance of *S. aureus*, and not some other change in the *∆aprA* mutant.

### Effect of the presence **of** *AprA* on the media and *S. aureus* silver tolerance

**Fig. 3.**
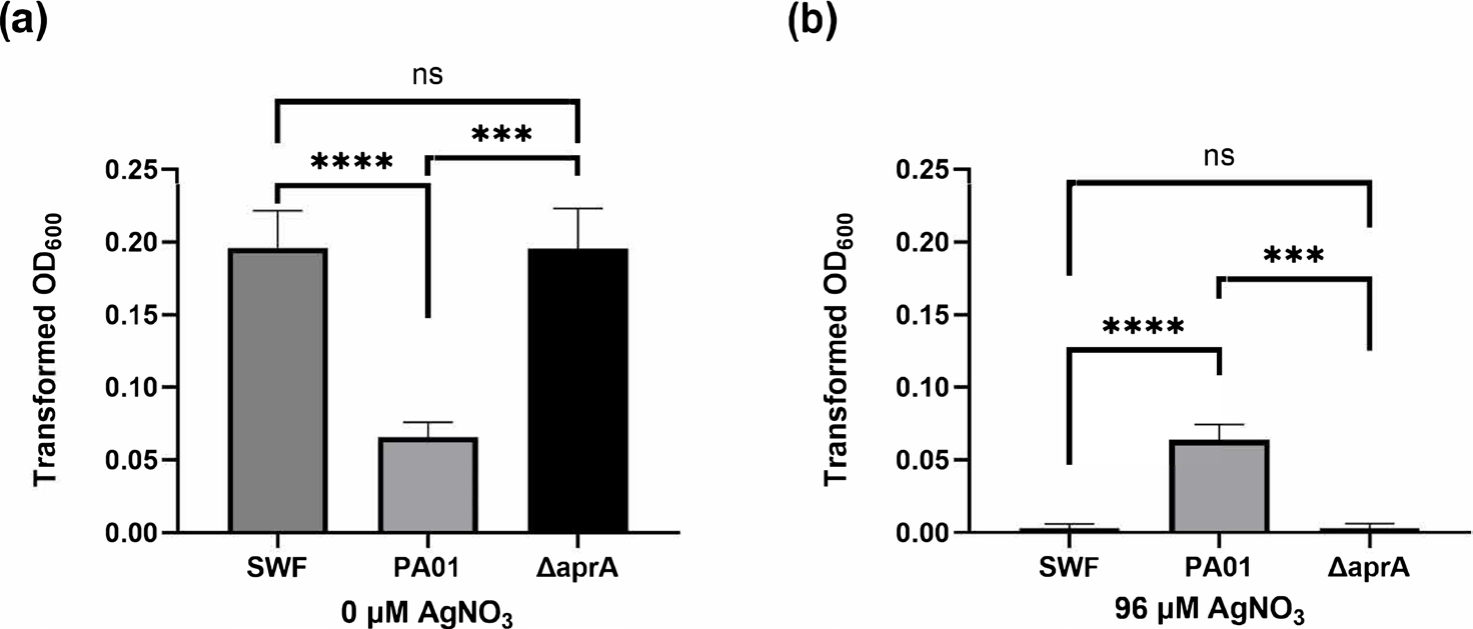
Evaluation of *S. aureus* grown in the presence of <3 kDa spent media from *P. aeruginosa* (PAO1), from the ∆*aprA* mutant or just SWF. (**a**) No exposure to metal or (**b**) exposed to 96 µM AgNO_3_. Cultures were grown for 24 h and the results of 3 biological replicates are shown with their sd. Transformed OD_600_ reflects that the OD_600_ of the culture at time zero was subtracted.

As AprA is a protease that could be acting on components of the SWF media, we next examined whether it was AprA itself that affects *S. aureus* or if it modifies the low-WM composition of the spent media. To do this, we grew *S. aureus* in spent media collected from both PAO1 and *∆aprA* strains of *P. aeruginosa*, each processed through a 3 kDa MW cutoff filter. Growth was assessed in the absence of antimicrobial metals to focus solely on the effects of the spent media. *S. aureus* grown in the *ΔaprA* spent media showed higher cell density as evaluated by absorbance at 600 nm than growth in the presence of WT PAO1 spent media (Fig. 3a). This suggests that there is a small MW compound that is antagonistic to *S. aureus* growth. The same compound could also be responsible for some of the resistance to silver (Fig. 3b).

### Pyocyanin and its effects on *S. aureus* growth and silver tolerance

AprA is linked to pyocyanin function [17] although it is not part of the direct biosynthesis *phz* pathway [18]. This redox-active phenazine is implicated in both growth-promoting and inhibiting activities [19]. These observations are similar to how we observed decreased growth of *S. aureus* in unchallenged conditions but increased under silver stress. Pyocyanin is also implicated in silver tolerance [20]. With this information, we extracted and quantified pyocyanin levels in spent media from the WT and the *ΔaprA* mutant. The WT strain produced a mean pyocyanin concentration of 4.4 µg ml^−1^ compared with the *ΔaprA* strain, which yielded only an estimated 0.17 µg ml^−1^. The *ΔaprA* spent media produced so little pyocyanin from the extraction that the concentration was difficult to accurately quantify. Regardless, these results indicate that the PAO1 strain of *P. aeruginosa* produces significantly (~25×) more (*P*<0.01) pyocyanin than the *ΔaprA* strain.

**Fig. 4.**
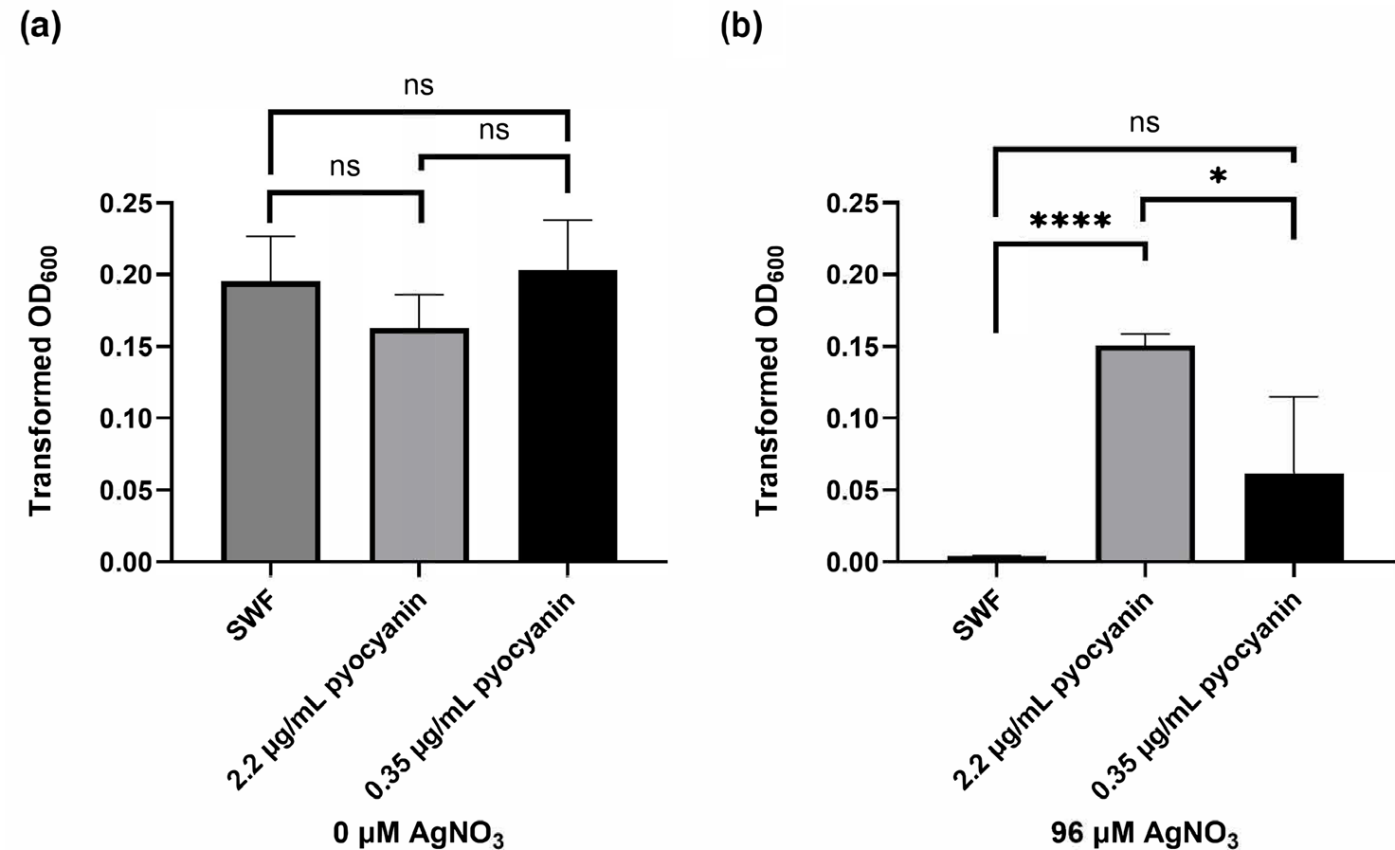
Growth of *S. aureus* in the presence of pyocyanin and silver. Mean OD at 600 nm (OD_600_) at time 24 h of *S. aureus* grown in the presence of pyocyanin concentrations in SWF, (**a**) without being exposed to metal and (**b**) exposed to 96 µM AgNO_3_. ‘SWF’ represents the SWF control without the addition of pyocyanin. The average and sd for each treatment were plotted from three biological trials.

As these results suggested that pyocyanin could be responsible for our observed effect on *S. aureus*, we tested whether pure pyocyanin could increase the silver tolerance of *S. aureus*. Two different concentrations of pyocyanin were tested: 0.35 µg ml^−1^, which mimics 8% spent media in the PAO1 WT strain (as in our previous experiments), and 2.2 µg ml^−1^, representing a higher pyocyanin concentration for comparison. Notably, high pyocyanin levels are shown to be enough on their own to protect *P. aeruginosa* from silver toxicity [20]. As controls, *S. aureus* growth was assessed in the absence of antimicrobial metals to observe the effects of pyocyanin alone. We noted that high pyocyanin levels lead to a slight inhibition of *S. aureus* growth (Fig. 4a), whereas increasing concentrations of pyocyanin protect *S. aureus* from silver challenge (Fig. 4b). The addition of pyocyanin to spent media from *P. aeruginosa* ∆*aprA* strain rescued the silver resistance for *S. aureus* (Fig. S6).

We also considered the possibility that the observed silver tolerance was caused by the secreted protease AprA digesting proteins in the SWF media into peptides and thus facilitating an Ag ion sequestration mechanism. As we specifically observed the increase in silver tolerance in SWF media [13], if this mechanism was occurring, it would be specific to proteins within the bovine calf serum or proteins specifically produced by *P. aeruginosa* upon response to the serum. We thus carried out a metabolomic comparison of extracellular metabolites and found a strong difference between LB and SWF (Figs S7 and S8). We then tested the hypothesis that there may be higher levels of AA from this proteolytic activity that would stimulate pyocyanin synthesis as suggested above, but our metabolomic data do not support this as no metabolite appeared to be drastically differentially accumulating in WT vs the ∆*aprA* strain (Fig. S8).

## Discussion

Co-virulence of *S. aureus* and *P. aeruginosa* has been shown to synergistically persist in chronic wound infections [21]. Since silver is often used in antimicrobial wound dressings, we have previously explored antimicrobial metal challenge to mixed species, observing remarkable changes in individual species tolerance compared to when they are grown on their own [22]. Investigating the explanation for these differences, we discovered that compounds below 3 kDa that are secreted by *P. aeruginosa* were found to increase the resistance of *S. aureus* to silver [13], but these compounds alone could not account for the full increase in observed resistance. In this follow-up study, we explore if there are any higher MW biomolecules such as secreted proteins from *P. aeruginosa* that could influence silver tolerance in *S. aureus*. This led to the discovery of AprA and its involvement with pyocyanin in silver tolerance.

AprA has been suggested to have a role in the biosynthesis of pyocyanin [17], a blue-coloured phenazine produced by *P. aeruginosa* [15]. Pyocyanin synthesis in *P. aeruginosa* involves complex biosynthetic pathways that are tightly regulated and interconnected with various cellular processes. This metabolite plays crucial roles in microbial physiology, virulence and interspecies interactions, having detrimental and even beneficial effects on microbial communities under different circumstances [23]. Phenazines are redox-active metabolites that can lower the membrane potential of cells they interact with, causing a reduction in proton pumping and subsequently cellular ATP levels [24]. Secretion of phenazines increases the tolerance to antibiotics of not only *P. aeruginosa* but also nearby cells of other species in a community [24,25]. Finally, interspecies redox behaviour was previously shown to affect silver resistance [26].

AA such as glycine, alanine, valine and tyrosine have been shown to promote pyocyanin production in *P. aeruginosa* [17]. Previously, we discovered that the AA serine and threonine and the *Pseudomonas* quinolone signal (PQS) were found to increase the resistance of *S. aureus* to silver [13]. In this earlier study, we considered that these biomolecules may be involved in reducing the bioavailability of Ag^+^ by acting as chelating metallophores, reducing the bioavailability of ligated silver. PQS likely contributes to this phenotype as it can interact with metals other than Fe [27], but its connection to the AprA phenotype will require further investigation. Metabolomic analysis of the spent media did not indicate any strong differences between the WT and ∆*aprA* strain, though our results indicate that the increase in silver tolerance is caused by multiple *P. aeruginosa* secretions, indicating that this is a complex phenotype.

Multiple proteases, particularly AprA, are involved in the virulence and immune system evasion of *P. aeruginosa* in chronic infections [28]. In addition to this role, AprA influences pyocyanin synthesis through unknown mechanisms [17]. As pyocyanin on its own can reduce Ag^+^ to Ag^0^ [20,26], which is far less toxic to bacteria, we now see the connection for AprA increasing the silver tolerance of *S. aureus* and that this could occur in mixed infection if treated with silver. Although our study has found a further explanation for how *P. aeruginosa* protects *S. aureus* from silver ion stress, the complete connection of the observations into a final biochemical mechanism still remains a mystery.

## Supplementary material

10.1099/mic.0.001596 Fig. S1.

## Abbreviations

IEC: ion exchange chromatography
LB: lysogeny broth
MW: molecular weight
PQS: *Pseudomonas* quinolone signal
SEC: size exclusion chromatography
SWF: simulated wound fluid
