# Supplementary material for: Role of AprA and pyocyanin from Pseudomonas aeruginosa on Staphylococcus aureus tolerance to silver
Source: Microbiology (Reading). 2025 Sep 3;171(9):001596. doi: 10.1099/mic.0.001596 (PMC12408190; doi:10.1099/mic.0.001596)
Supplement: Fig. S1. [file mic-171-01596-s001.pdf]

Supplementary Material

Role of AprA and Pyocyanin from *Pseudomonas aeruginosa* on *Staphylococcus aureus* Tolerance to Silver

Jakob Gorodetsky[1], Nadia Monich[1], Raymond J. Turner[1], Omid Haji-Ghassemi,[1#] Sean C. Booth[2#]

Table S1: Proteomics identification results[1] of activity fraction

Name	Putative function	UNIPROT
1) Pseudolysin/Elastase (lasB)	metalloendopeptidase (requires Zn and Ca) that cleaves elastin and collagen	P14756
2) Serralysin (aprA)	alkaline protease which binds Zn and Ca	Q03023
3) Lysyl Endopeptidase (prpL)	lysine specific endoprotease	Q9HWK6
4) Protease (lasA)	has proteolytic and elastoltic activity also degradespeptidoglycan Zn as cofactor	P14789
5) 60 kDa chaperonin (groEL)	Stress response protein foldase	P30718
6) Aminopeptidase (lieA)	inferred metalloprotease	A0A069QAR2
7) transporter (sstT)	Ser/Thr amino acid Na exchanger	Q9I273

[1] Rank list of proteins based on 99% significance cut off using Mascot software. Eleven additional proteins were found but not listed and not considered further as they only had one peptide match.

AprA enzymatic Assays (Azocasein digestion Assay).

Supplementary experiments using azocasein digestion assay to evaluate protease activity within spent media fractions.

Method

Azocasein Digestion Assay. Protease activity was assessed on the PA01 and *ΔaprA* spent media fractions using proteolytic activity assay adapted from [17]. 50 µL of the solution to be assessed was mixed with 50 µL of 5 mg/mL azocasein (Sigma-Aldrich) substrate dissolved in 100 mM Tris-Cl, 5 mM $CaCl_2$, pH 8.0 and incubated at 37°C for one hour. After incubation, 200 µL of 10% trichloroacetic acid was added and the samples were incubated at 4 °C for 15 minutes. The samples were then centrifuged at 12,000 rpm at 4 °C for 5 minutes. 150 µL of each sample supernatant was added to a 96 well plate containing 150 µL of 525 mM NaOH in each well where the sample would be added. The absorbance of the solutions in the wells of the plate was measured at 450 nm. Negative controls where 50 µL of the filtered spent media fractions that were heat inactivated at 100 °C for 10 minutes prior to azocasein addition, and were utilized as a baseline for the absorbances to account for non-specific casein cleavage.

Results

Figure S1 shows fractionation of cell extracts grown in terrific broth (LB + glycerol). The data reflects that only *P. aeruginosa* WT and not the Δ*aprA* strain has azoczasien protease activity and this activity is still presence in high ammonium sulphate concentrations. Figure S2 reflects the protease activity after the ammonium sulphate has been dialyzed out. This finding demonstrates that there are other proteases present in the Δ*aprA* strain's spent media, but they are not active in high salt concentrations.

The difference in the secreted proteases with media is reflected in Figure S3 where the *P. aeruginosa* was grown in SWF. In this case after dialysis the Δ*aprA* strain has little protease activity whereas the WT does suggesting this media leads to other proteases being secreted beyond AprA. Figure S3 also shows that *P. aeruginosa* grown under different silver stresses all have similar protease activity levels and thus *aprA* levels are not dependent on silver exposure.

This supporting data demonstrates that the AprA is the primary protease active when grown in SWF and that this activity is not dependent on growth in the presence of silver nitrate.

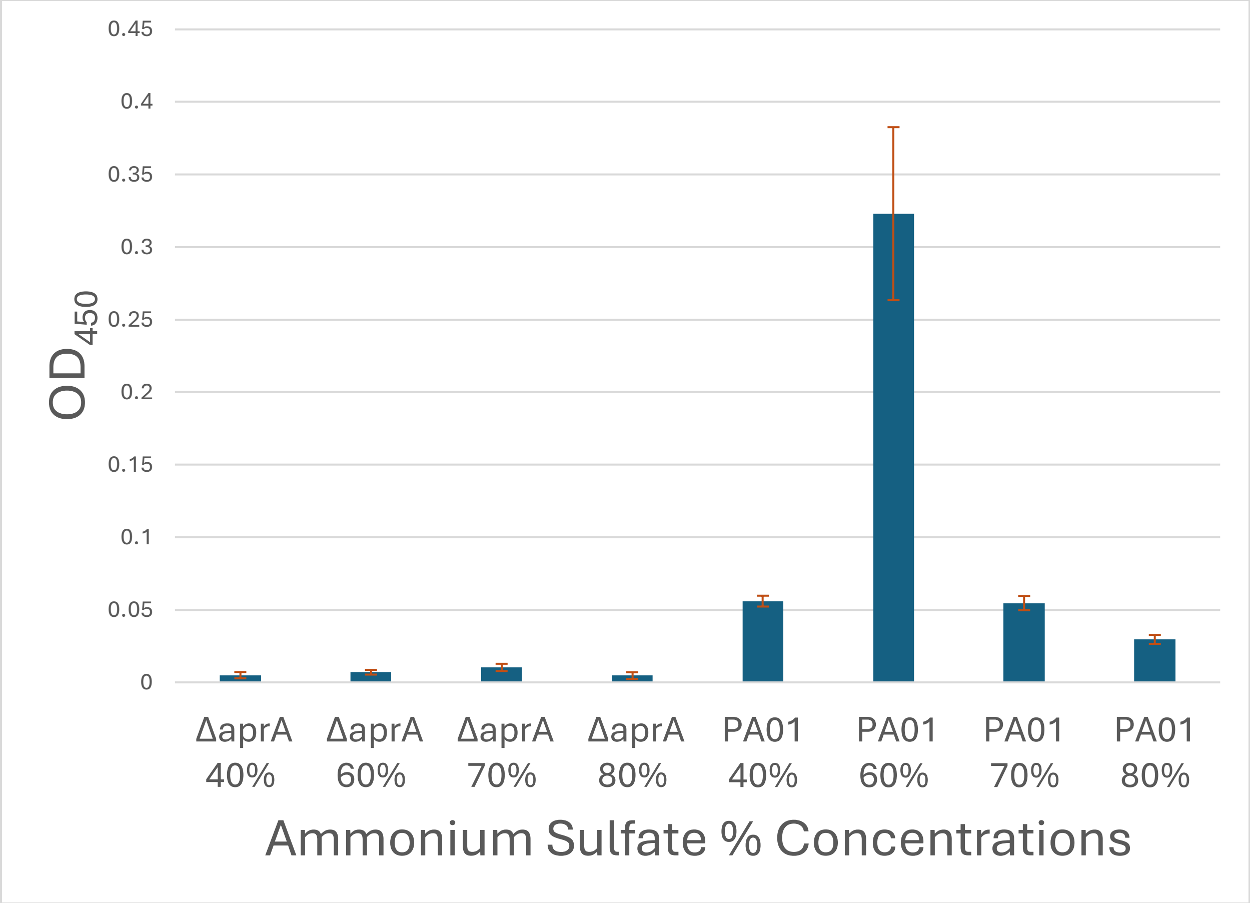

Figure S1: Azocasein digest absorbances from spent media precipitated with ammonium sulfate (Strains grown in terrific broth). This assay was done pre-dialysis, indicating that AprA precipitates best at 60% ammonium sulfate and that it is active in high salt environments.

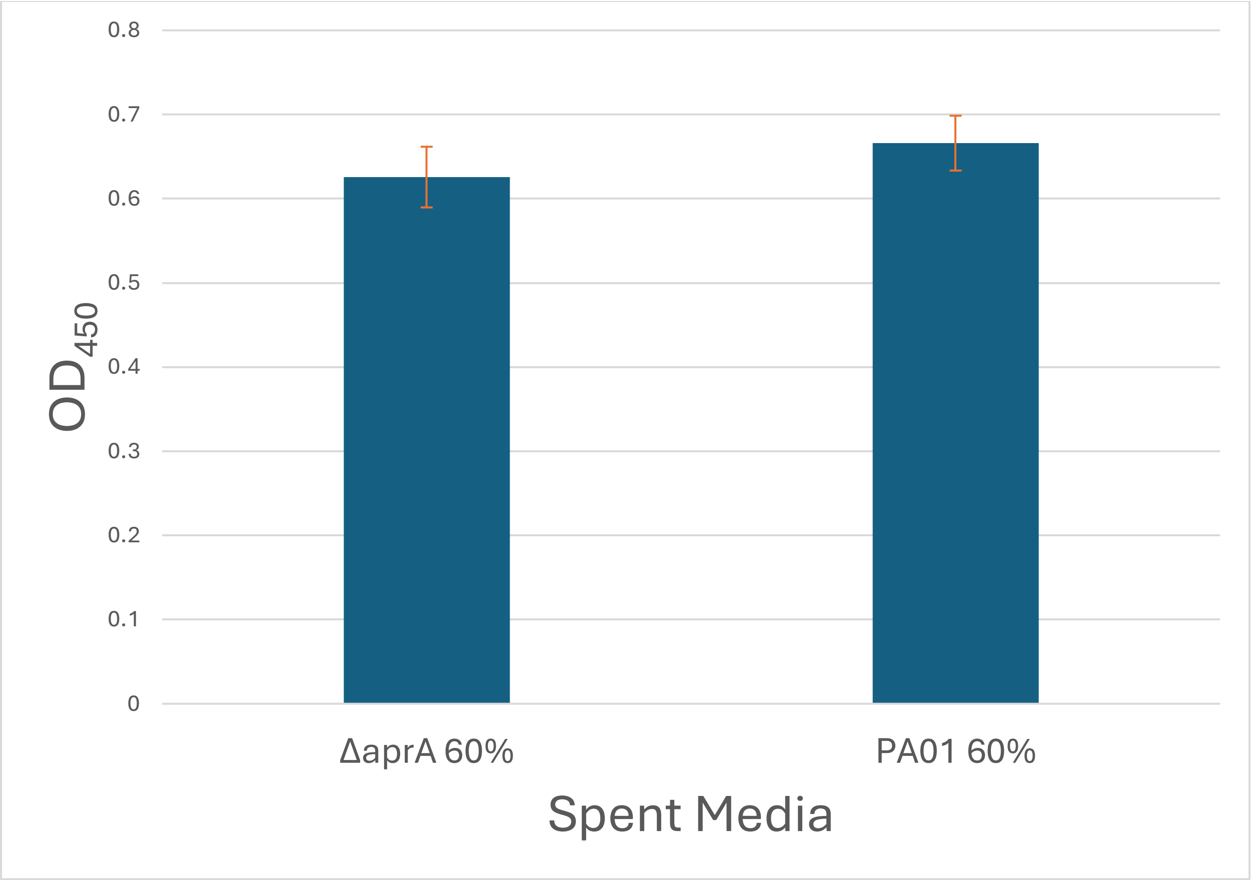

Figure S2. Azocasein digest absorbances from spent media precipitated with ammonium sulfate (grown in terrific broth). This assay was done post-dialysis, indicating that protease activity returns to the *ΔaprA* after the salt has been dialyzed out.

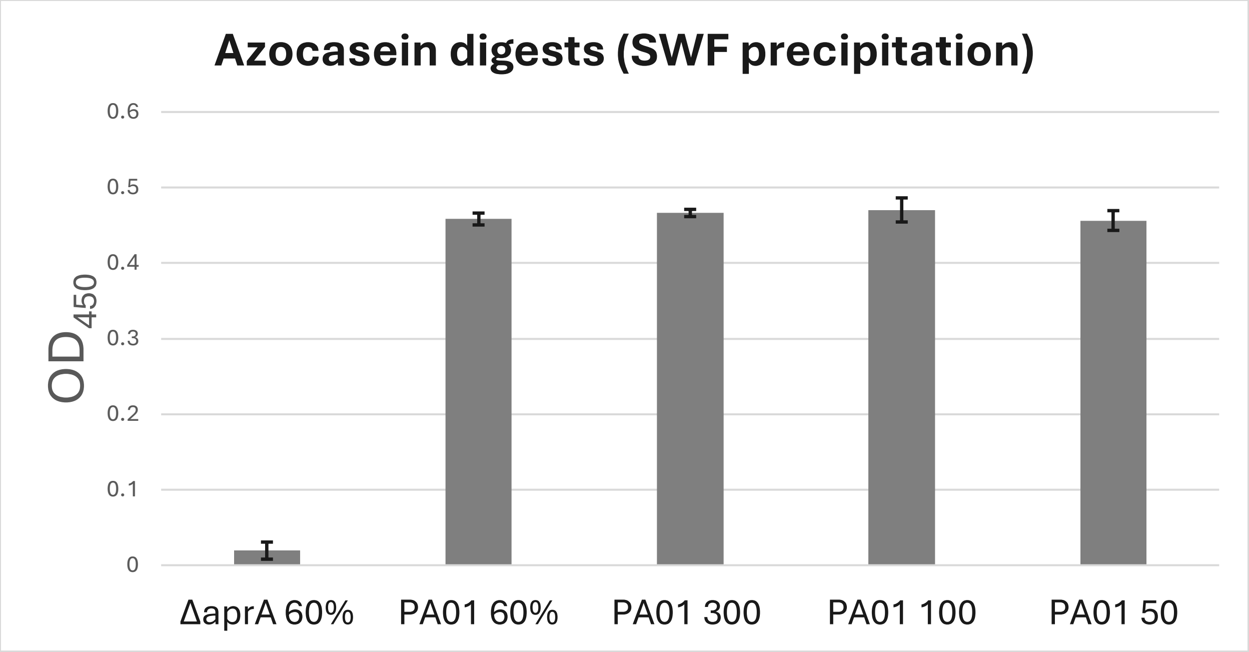

Figure S3: Azocasein digest absorbances from spent media precipitated with ammonium sulfate (grown in SWF). This assay was done post-dialysis, indicating that protease activity does not return to *ΔaprA* after the salt has been dialyzed out, being different than TB where it did return. Also tested the precipitates at different $AgNO_3$ concentrations (300, 100 and 50 micromolar), indicating that silver has no effect on AprA activity.

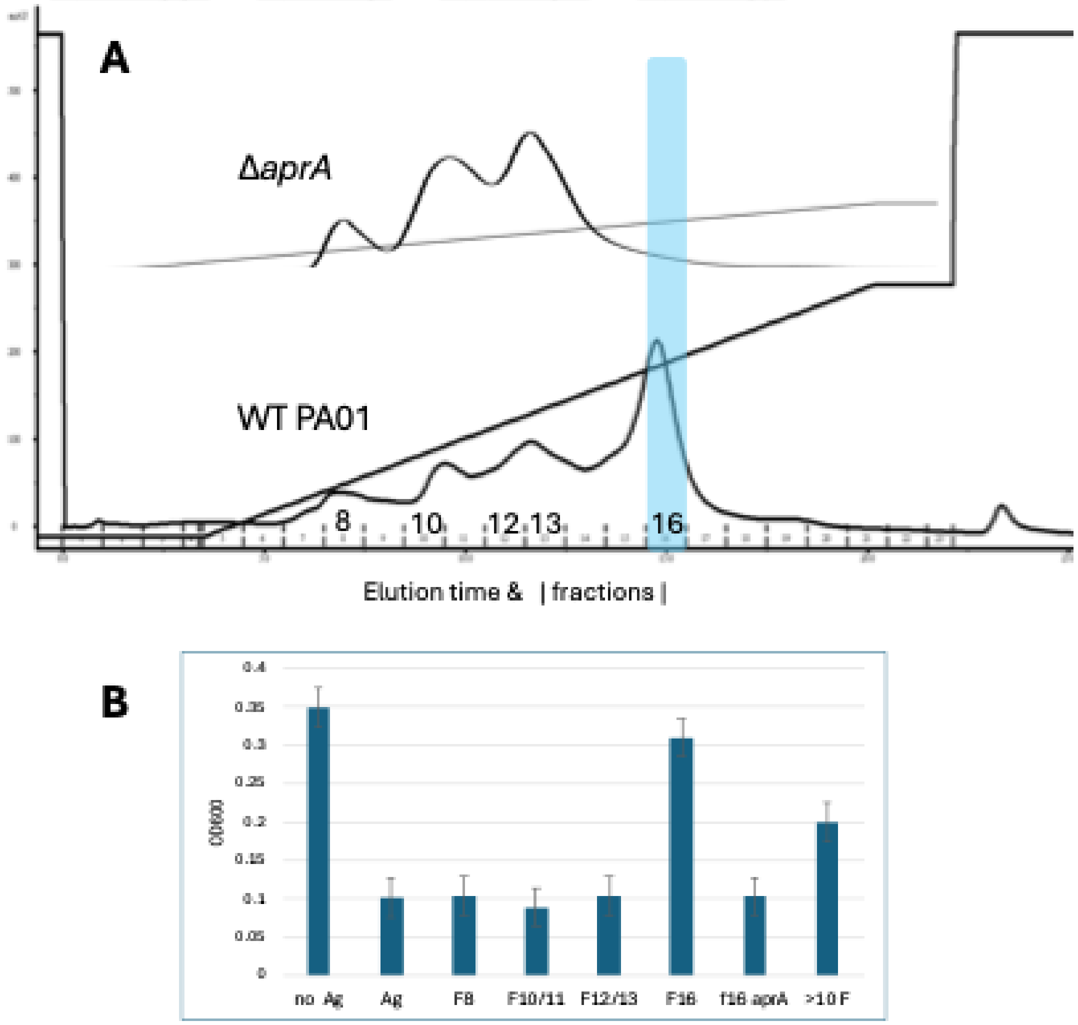

Figure S4. A: Chromatogram from MonoQ column. Loading onto column the highest activity fraction from ammonium sulphate precipitation. The Lower chromatogram is from Wild type PA01 and for comparison loading the same ammonium sulphate precipitation fraction from treatment of the spent media from *P. aeruginosa* Δ*aprA*. The light blue bar defines Fraction 16. **B:** The bioassay of growth of *S. aureus* in the presence of 50 µM $AgNO_3$ with media augmented with different fractions from the chromatogram. No Ag is growth control without Ag challenge, >10F is the greater than 10kDa fraction from PAO1 spent media which would contain AprA.

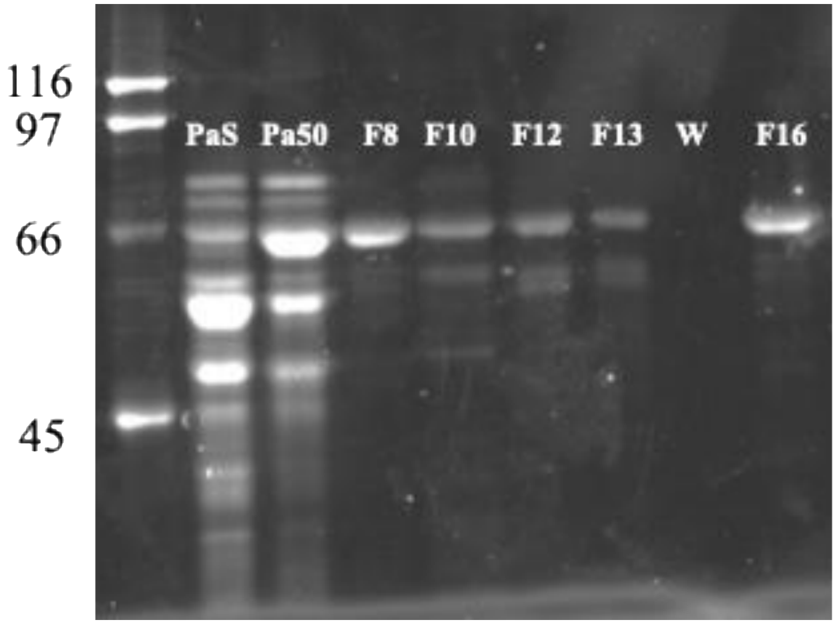

Figure S5: 10% SDS-PAGE of protein fractions (F) from MonoQ column. Densitometer analysis of fraction 16 (F16) suggest >85% pure. kDa molecular weights of the size standards are indicated left of bands in first lane. Fraction ‘W’ is the flow through wash fraction from end of column run. Gel is fluorescence image from in gel staining defined method providing greater detection level (Ladner, et al., (2004) Visible Fluorescence Detection of Proteins in Polyacrylamide Gels without staining. *Anal. Biochem*. 326;13-20).

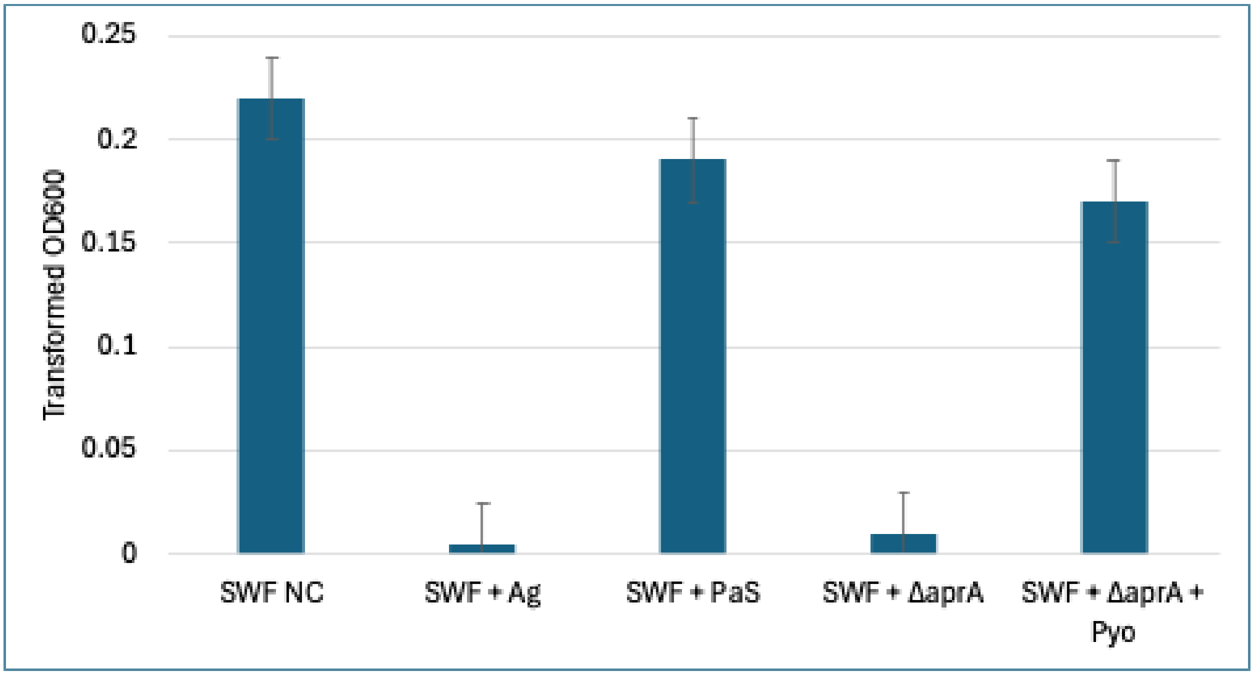

Figure S6. Exogenous pyocyanin addition rescues Δ*aprA* phenotype for S. aureus survival to challenge at 90 µM. SWF NC; growth of S. aureus in SWF with no $AgNO_3$ challenge, SWF + Ag growth of S. aureus in SWF with $AgNO_3$ challenge. SWF+PaS; addition of *P. aeruginosa* PA01 spent media, SWF +Δ*aprA*, addition of spent media from Δ*apr* strain of *P. aeruginosa*. SWF +Δ*aprA* + Pyo, addition of spent media from Δ*apr* strain of *P. aeruginosa* augmented with pyocyanin at 2 µg/mL.

Extracellular Metabolomics Investigation

Cultures of wild type PA01 and Δ*aprA* strain of *P. aeruginosa* were grown in LB media or simulated wound fluid (SWF) in 2 mL volume at 37 °C overnight and this culture was normalized to same cell density (OD600) and used as a 2% inoculant of a 1 mL of media and incubated 37 °C 200 rpm shaking for 8 hours. Three biological replicates were used. 500 µL of culture was removed and centrifuged at 10,000g for 10 min. 20 µL of supernatant was added to 380 µL of 50% methanol:ddH_2O. This sample was then supplied to the Calgary Metabolomics Research Facility for LC-MS-MS for metabolite detection. A second 50 uL sample of the culture was taken to establish cell density. MAVEN (El-MAVEN v0.12.0) software was utilized to analyze the acquired data. In house R-scripts were used for PCA and heat map plots.

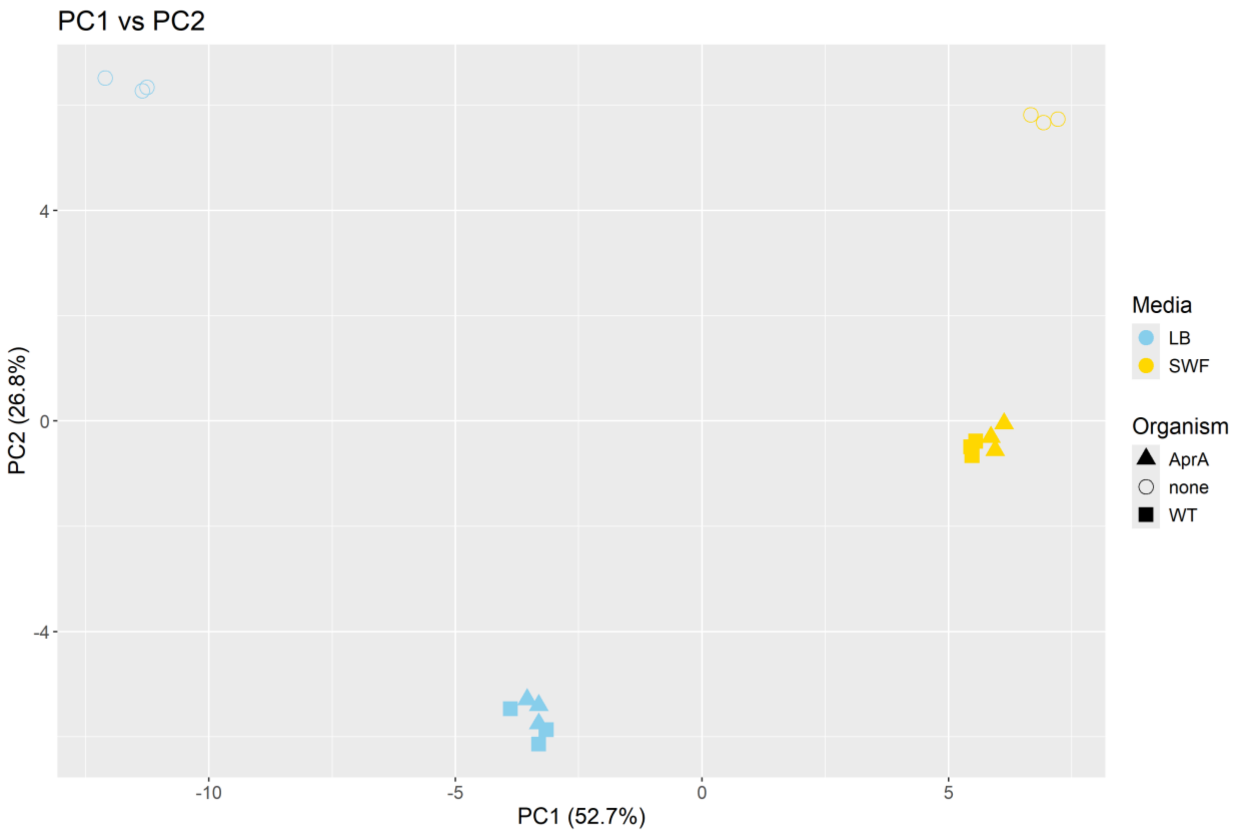

Figure S7: Principle component analysis of extracellular metabolites from cultures of type PA01 and Δ*aprA* strain of *P. aeruginosa* in two different media. Data separates based on media type. Only a minor separation is seen in SWF between WT and mutant cultures. For organism "open" are the media controls with no bacteria added (indicated as circles on the plot).

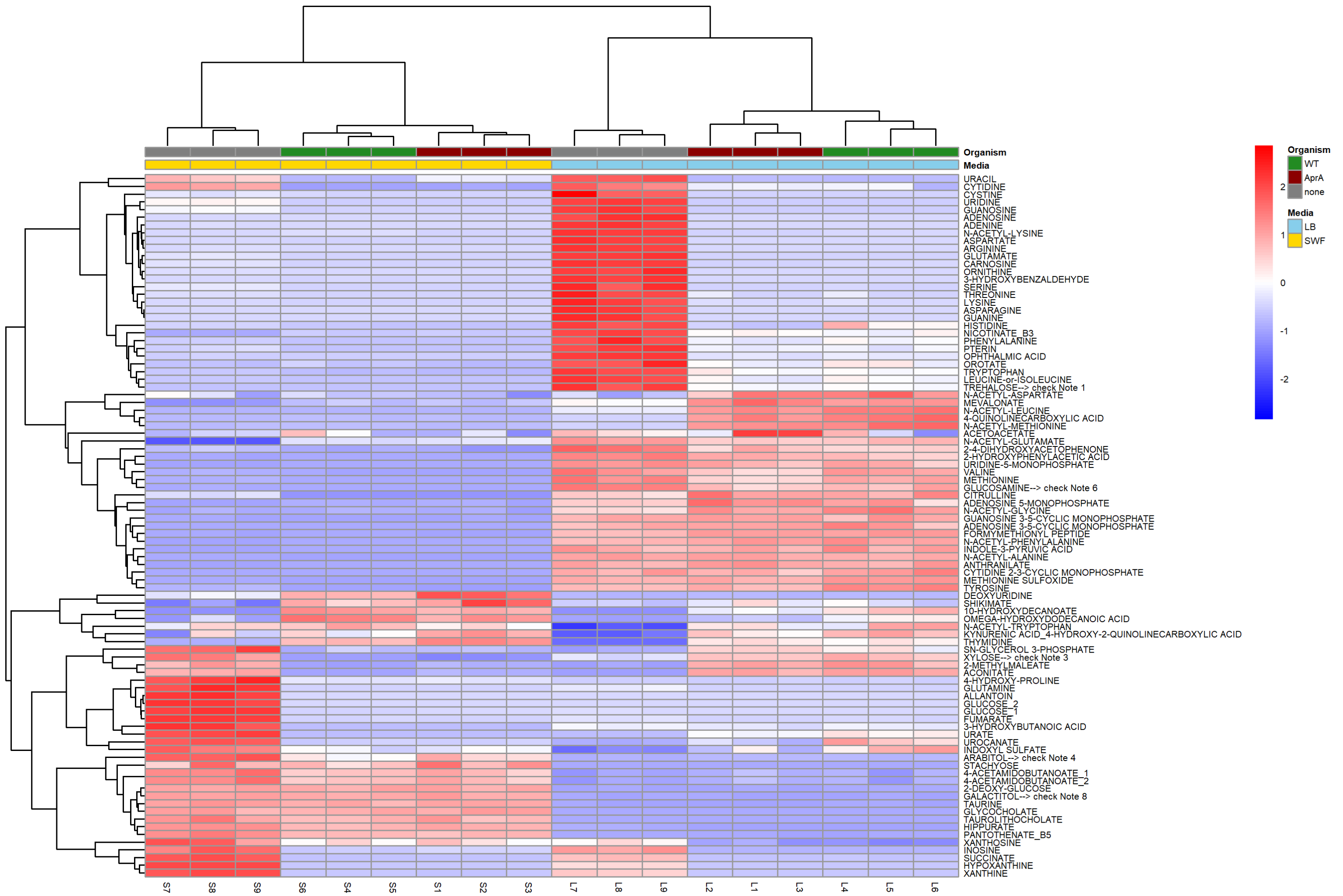

Figure S8. Heat map of hierarchal clustering of extracellular metabolites from cultures of wild-type PA01 and Δ*aprA* strain of *P. aeruginosa* grown either on LB or SWF medium. Metabolites extracted from media with no bacteria are also shown (none). The color coding map is showing z-scores, normalized by row (compound, in this case) and provides measure of variability within the same compound.
